# The syndemic effects of adverse mental health conditions and polysubstance use on being at risk of clinical depression among marginally housed and homeless transitional age youth living in San Francisco, California

**DOI:** 10.1371/journal.pone.0265397

**Published:** 2022-03-17

**Authors:** Jennifer P. Jain, Glenn-Milo Santos, Jennifer Hao, Adam Leonard, Aaron M. Miller, Yvette P. Cuca, Carol Dawson-Rose

**Affiliations:** 1 Department of Community Health Systems, University of California, San Francisco, California, United States of America; 2 Department of Psychiatry and Behavioral Sciences, University of California, San Francisco, California, United States of America; 3 School of Medicine, University of California, San Francisco, California, United States of America; University of Toyama: Toyama Daigaku, JAPAN

## Abstract

The objective of this study was to identify the correlates of being at risk of clinical depression and examine the role of syndemic factors among marginally housed and homeless transitional age youth (TAY). From 2017–2018, 100 TAY between the ages of 18 and 24 in San Francisco were recruited from Larkin Street Youth Services into a cross-sectional study. Participants completed surveys on mental health, substance use, and HIV risk behaviors. A syndemic score ranging from 0–3 was calculated by summing dichotomous measures of moderate or severe anxiety in the past two weeks, PTSD symptoms in the past month and polysubstance use in the past three months. We used modified Poisson regression with robust error variances to identify the correlates of being at risk of clinical depression in the past week, all primary effects measures were modeled separately. Among 100 participants, the average age was 21 (SD = 1.7), 67% were male, 38% were Multiracial, 54% identified as gay, lesbian, bisexual or pansexual, 13% were unstably housed, 50% were homeless and 23% were living with HIV. The majority (74%) were at risk of clinical depression, 51% had symptoms of moderate or severe anxiety, 80% exhibited symptoms of PTSD and 33% reported polysubstance use. After controlling for age in years, gender, race/ethnicity and sexual orientation, factors independently associated with being at risk of clinical depression were; symptoms of moderate or severe anxiety (adjusted risk ratio [aRR] = 1.62, 95% confidence interval [CI] = 1.23–2.12, *P*<0.001), symptoms of PTSD (aRR = 3.78, 95% CI = 1.58–9.04, *P* = 0.003), polysubstance use (aRR = 1.33, 95% CI = 1.06–1.68), *P* = 0.012), an increasing syndemic score (aRR = 1.40, 95% CI = 1.21–1.62), *P*<0.001), and having one, two or three syndemic factors (compared to none); (aRR = 2.68, 95% CI = 1.08–6.65, *P* = 0.032), (aRR = 3.24, 95% CI = 1.24–7.83, *P* = 0.003) and (aRR = 3.97, 95% CI = 1.65–9.52, *P* = 0.002), respectively. Integrated behavioral health models that treat co-occurring disorders simultaneously are needed to reduce syndemic risk among marginally housed and homeless TAY.

## Introduction

Approximately 1 in 10 transitional age youth (TAY) between the ages of 18 to 24 experience homelessness every year in the United States (US), representing 3.5 million youth annually [1]. Prior research has consistently demonstrated an increased prevalence of mental health disorders among this population, including high rates of depression, anxiety, substance use and post-traumatic stress disorder (PTSD) [2–5]. Overall, the lifetime prevalence of psychiatric conditions among youth experiencing homelessness is twice that of their stably-housed peers [6]. Further, unstably housed TAY experience high rates of co-occurring substance use disorders with either anxiety, or affective disorders including major depressive disorder or bipolar disorder, a phenomenon known as dual diagnosis [7].

Among homeless TAY with multiple comorbid psychiatric conditions, major depressive disorder is the most common diagnosis, affecting anywhere from 41% to 73% of these individuals [7–9]. Studies have also demonstrated that suicidal ideation and attempt are markedly higher among unstably housed TAY. For instance, 40% to 80% of homeless youth report suicidal ideation, and 23% to 67% report at least one prior suicide attempt [3, 6, 8, 10]. In San Francisco, one study found that the mortality rate among homeless TAY was 10-fold higher than stably-housed, age-matched peers, largely due to increased deaths from suicide and conditions associated with severe substance use disorder [11].

The causes of homelessness among many TAY include violence and abuse, including physical, sexual or emotional abuse experienced in homes of origin [12]. Following the onset of homelessness, many TAY continue to encounter victimization and abuse. For instance, one study found that 83% of youth reported one or more instances of physical or sexual victimization while living on the streets, which contributes to poor mental health outcomes [13]. Experiences of violence and victimization vary by sexual orientation [1, 5]. For instance, lesbian, gay, bisexual or transgender (LGBT) youth are more likely to face sexual victimization and harassment from police compared to their counterparts, increasing their vulnerability to developing mental health disorders such as anxiety, PTSD and depression [14]. Further, other homeless LGBT TAY report higher rates of depression and suicidality compared to their heterosexual and cisgender peers [15].

While the high prevalence of various mental health disorders among unstably housed TAY is well-established in the literature, few studies to our knowledge have employed a syndemic approach to understand how the co-occurrence of anxiety, PTSD symptoms and polysubstance use impact depression. Originally conceived in the early years of the HIV/AIDS pandemic by Merrill Singer, Syndemic Theory is a model for conceptualizing how two or more co-occurring health conditions can interact *synergistically* within a specific population and social context to mutually increase the overall burden of deleterious health outcomes [16–18]. Notably, the term ‘syndemics’ is not a synonym for comorbidities, but rather a phenomenon that develops under the co-occurrence of various adverse socio-structural conditions which in turn increases the risk of developing negative health outcomes. Since its conception, Syndemic Theory has been widely applied in medical, anthropological, and public health research to better understand the impact of disease clustering [19]. Leveraging this framework to understand the synergistic effects of anxiety, PTSD and polysubstance use on depression among unstably housed TAY may help inform service delivery methods to improve the overall health of this marginalized population.

To address this knowledge gap, we studied marginally housed and homeless TAY between the ages of 18 and 24 in San Francisco, California (CA) to identify the prevalence and correlates of being at risk of clinical depression. Additionally, we employed a syndemics framework [17] to examine whether the co-occurrence or clustering of multiple (i.e., two or more) adverse psychosocial factors, including symptoms of moderate or severe anxiety, symptoms of PTSD and polysubstance use, had a synergistic effect on being at risk of clinical depression among high-risk youth. We hypothesized that the prevalence of being at risk of clinical depression would be high and associated with a greater number of syndemic factors. As such, this study fills an important gap in research on how syndemic experiences fuel inequalities in psychological and socio-behavioral outcomes among marginally housed and homeless TAY in San Francisco, CA.

## Methods

### Study design and eligibility

We utilized baseline data from a Substance Abuse and Mental Health Services Administration (SAMHSA) funded study designed to assess mental health, substance use and HIV risk behaviors among marginally housed and homeless TAY at Larkin Street Youth Services in San Francisco, CA. Participants were considered eligible if they were: 1) between the ages of 18 and 24, and 2) clients of Larkin Street Youth Services. Larkin Street Youth Services is a community-based organization [20] that provides a wide variety of services, including housing, case management, education and employment training programs, and medical care for marginally housed and homeless youth in San Francisco.

### Recruitment

From May 2017 through April 2018, 100 TAY were recruited from various Larkin Street Youth Service sites, including transitional housing sites, one of which was designed for youth living with HIV, and drop in-centers. Recruitment strategies involved posting recruitment flyers, giving presentations at community meetings for Larkin Street clients, and coordinating closely with Larkin Street staff to recruit participants.

### Study procedures

Surveys were administered by trained interviewers with extensive experience working with high-risk TAY. All interviews were conducted in a private setting and lasted approximately 90 minutes. Data were collected using a computer assisted survey information collection (CASIC) method administered on iPads. Participants received a $30.00 drugstore gift card for their participation. Participation in the survey had no bearing on individuals’ ability to obtain services at Larkin Street.

### Ethical considerations

All participants provided written informed consent at baseline. All study procedures were approved by the Institutional Review Board at the University of California, San Francisco (IRB Protocol #: 17–21673).

## Measures

### Outcome

The primary outcome of interest was being at risk of clinical depression. We used the 20-item Center for Epidemiologic Studies Depression Scale (CESD-20) to measure depressive symptoms in the past week (Cronbach’s alpha = 0.87) [21]. Participants were asked to record how often they experienced different emotions in the past week using the following Likert-scale: 0 = Rarely or none of the time, 1 = Some or little of the time, 2 = Moderately or much of the time, and 3 = Most or all of the time. Scores were summed to create a total score ranging from 0–60 and a cutoff of 16 was used to create a dichotomous measure of being at risk of clinical depression (yes/no).

### Sociodemographics

Data on age in years, race/ethnicity (White, Black or African American, Multiracial, or other) and gender (male, female, or “other” with an open field option) were collected. Sexual orientation was measured by creating a dichotomous variable for those who identified has being heterosexual versus gay, lesbian, bisexual, or pansexual (yes/no). Participants were asked to describe where they live by selecting one of the following responses: 1) In my own apartment, 2) In a relative’s home, 3) In a group home, 4) In a campus/dormitory housing, 5) In a foster care, 6) homeless or in a shelter, and 7) other. From these responses, a categorical measure of housing stability was created with the following categories; stably housed (i.e., living in an apartment or on campus), unstably housed (i.e., living in a relative’s home or group home) and homeless or living in a shelter. Data on ever having been incarcerated for three or more days (yes/no) and self-reported HIV serostatus (positive, negative/unknown) were also collected.

### Mental health exposures

Symptoms of post-traumatic stress disorder (PTSD) in the past month were assessed via the 20-item PCL-5 (DSM-5 PTSD checklist) (Cronbach’s alpha = 0.95) [22]. Total scores range from 0–80, and a standard cutoff of 33 was used to create a dichotomous measure of PTSD symptoms (yes/no). We used the Generalized Anxiety Disorder 7-item (GAD-7) (Cronbach’s alpha = 0.89), to measure symptoms consistent with anxiety in the past two weeks [23]. Total scores range from 0–21 and designated cutoffs for minimal (0–4), mild (5–9), moderate (10–14) and severe (15+) were used to create a categorical measure of symptoms of anxiety. A dichotomous measure of symptoms of moderate or severe anxiety (yes/no) was created for those with scores of 10 or greater. We measured any exposure to traumatic events prior to the age of 18 using the Adverse Childhood Experiences (ACEs) instrument [24, 25]. Traumatic events assessed included experiences of emotional, physical and sexual abuse. A cutoff of 4 or more was used to create an indicator variable for greater adverse childhood experiences (yes/no). These instruments were not used as diagnostic tools, they were used to evaluate the presence of symptoms consistent with the mental health conditions assessed to decide whether further psychiatric evaluation was needed.

### Substance use

Drug and alcohol use were assessed using the NIDA-Modified ASSIST [26]. Participants were asked if they used any of the following drugs: cannabis, cocaine, prescription stimulants, methamphetamine, inhalants, sedatives, hallucinogens, street opioids, prescription opioids or other drugs in the past three months (yes/no). Consistent with prior research on polysubstance use and syndemics [27], polysubstance use was defined as using three or more of the drugs listed above in the past three months (yes/no). Binge drinking in the past year (yes/no) was measured using standard cutoffs (i.e., 5 or more drinks for males and 4 or more drinks for females on the same day/single occasion).

### Syndemic risk

Consistent with other research on syndemics [28, 29], a composite syndemic score ranging from 0–3 was created by summing dichotomous measures of; moderate or severe anxiety, PTSD symptoms and polysubstance use. We selected these factors based on a priori hypotheses related to broader mental health disease clustering and their confirmed association with the outcome of interest (being at risk of clinical depression). Factors that were not significantly (e.g., *P*≤0.05) associated with being at risk of clinical depression in bivariate analyses were not included the syndemic score. A nominal syndemic variable was also created to identify those with zero, one, two or three syndemic factors.

## Statistical analysis

### Descriptive statistics

We used descriptive statistics to describe the study sample and examine the prevalence of various mental health factors, substance use and sociodemographic characteristics. We generated frequencies, percentages and depending on distributional assumptions for continuous data means, standard deviations (SD) or medians and interquartile ranges (IQR).

We calculated pairwise correlation coefficients and corresponding p-values to estimate the level of clustering among the syndemic factors included in this study and to ensure they are true syndemic factors. Then, we examined the prevalence of being at risk of clinical depression by number of syndemic factors (i.e., for those with zero, one, two and three syndemic factors). We also graphed one’s depression score (0–60) by the number of syndemic factors (0–3).

### Modified poisson regression

We used modified Poisson regression with robust error variances to estimate the relative risk of being at risk of clinical depression by various sociodemographic, mental health, substance use and syndemic factors. Per Zou and colleagues recommendation, this method was used to yield more precise estimates including, smaller confidence intervals [30]. Each primary exposure that was significantly (i.e., *P*≤0.05) associated with being at risk of clinical depression at the bivariate level including: symptoms of moderate or severe anxiety, symptoms of PTSD, polysubstance use, the composite syndemic score and the nominal syndemic variable, was explored further in multivariable Poisson regression models. As recommended by Westreich and colleagues [31], each primary effect measure was modeled separately in order to yield total effect estimates and avoid multicollinearity. Multivariable models controlled for the following correlates of depression: age in years, gender, race/ethnicity and sexual orientation. Interactions were tested between each primary exposure and sexual orientation. All analyses were performed using Stata 16.1.

## Results

Among a total of 100 participants, the average age was 22 (SD = 1.7), 67% were male, 38% were Multiracial, 28% were Black, 22% were White and 12% identified as other or declined to state their race/ethnicity. Over half (54%) identified as gay, bisexual or pansexual, 13% were unstably housed and 50% were homelessness. Nearly a quarter (23%) were living with HIV and almost a third (29%) had ever been incarcerated for at least three days (Table 1).

**Table 1:** Sociodemographic, substance use and mental health characteristics among marginally housed and homeless transitional age youth in San Francisco, CA (N = 100).

*Variable*	n (%)
Mean age in years (SD)	22 (1.7)
*Race/ethnicity*	
Multiracial	38 (38)
Black	28 (28)
White	22 (22)
Other or declined to state	12 (12)
*Gender*	
Male	67 (67)
Female	28 (28)
Other	5 (5)
*Sexual orientation*	
Gay, lesbian, bisexual or pansexual	52 (52)
Heterosexual	44 (44)
Unsure	4 (4)
*Housing status*	
Stably housed	37 (37)
Unstably housed^1^	13 (13)
Homeless	50 (50)
Living with HIV	23 (23)
Ever incarcerated for 3 or more days	29 (29)
*Mental Health*	
At risk of clinical depression^2^	73 (74)
Symptoms of minimal anxiety^3^	23 (23)
Symptoms of mild anxiety	26 (26)
Symptoms of moderate anxiety	25 (25)
Symptoms of severe anxiety	25 (25)
Symptoms of moderate or severe anxiety	50 (51)
Symptoms of post-traumatic stress disorder^4^	79 (80)
High exposure to traumatic events prior to age 18^5^	76 (77)
*Substance Use Past 3 Months*	
Cannabis	46 (47)
Cocaine	32 (33)
Prescription stimulants	10 (10)
Methamphetamine	28 (29)
Inhalants	7 (7)
Sedatives	19 (19)
Hallucinogens	25 (26)
Street opioids	12 (12)
Prescription opioids	13 (13)
Other^6^	3 (3)
Polysubstance use^7^	32 (33)
*Alcohol Use*	
Binge drinking past year^8^	69 (70)
*Syndemic variables*	
Median continuous syndemic score (IQR)	2 (1, 2)
Number of syndemic factors	
0	18 (18)
1	22 (22)
2	37 (38)
3	21 (21)

Notes:

^1^Participants were considered unstably housed if they were living with a relative or in a group home.

^2^The CESD-20 was used to measure being at risk of clinical depression using a cutoff of 16.

^3^The GAD-7 was used to measure different levels of anxiety.

^4^The PCL-5 was used to measure PTSD symptoms using a cutoff of 33.

^5^The ACE’s instrument was used to measure high exposure to traumatic events prior 18 using a cutoff of 4.

^6^ Included: “belladonna, Hawaiian plant, native ritualistic sacraments and other psychedelics”.

^7^ Polysubstance use is defined as using three or more drugs (excluding alcohol).

^8^ Binge drinking was defined as ≥5 drinks for males and ≥4 drinks for females in the same day.

Some percentages are based on denominators smaller than the total N, this is due to missing data.

Frequencies were rounded up to the nearest whole number if the digit in the tenths place was ≥5.

The median CESD score was 25.3 (SD = 12.2) and 74% met symptom criteria for clinical depression evidenced by having a score of 16 or greater. Anxiety symptoms ranged from 23% with minimal, 26% with mild, 25% with moderate, 25% with severe and 51% with moderate or severe anxiety symptoms. The mean PCL-5 score was 54.6 (SD = 21.9) and 80% had a PCL-5 score of 33 or greater which is indicative of probable PTSD (not diagnostic PTSD). The mean ACEs score was 5.8 (SD = 2.6) and 77% had an ACEs score of 4 or more which is indicative of significant abuse, neglect and/or household dysfunction (Table 1).

The prevalence of substance use in the past three months was: 47% for cannabis, 33% for cocaine, 29% for methamphetamine, 26% for hallucinogens, 19% for sedatives, 13% for prescription opioids, 12% for street opioids, 10% for prescription stimulants, 7% for inhalants, and 3% reported the use of other substances, including: “belladonna”, “Hawaiian plant”, “native ritualistic sacraments”, “mushrooms” and “other psychedelics”. A third (33%) engaged in polysubstance use (i.e., the use of three or more drugs) in the past three months. Over two-thirds (70%) reported binge drinking in the past year (Table 1).

### Syndemic factors

**Fig 1:**
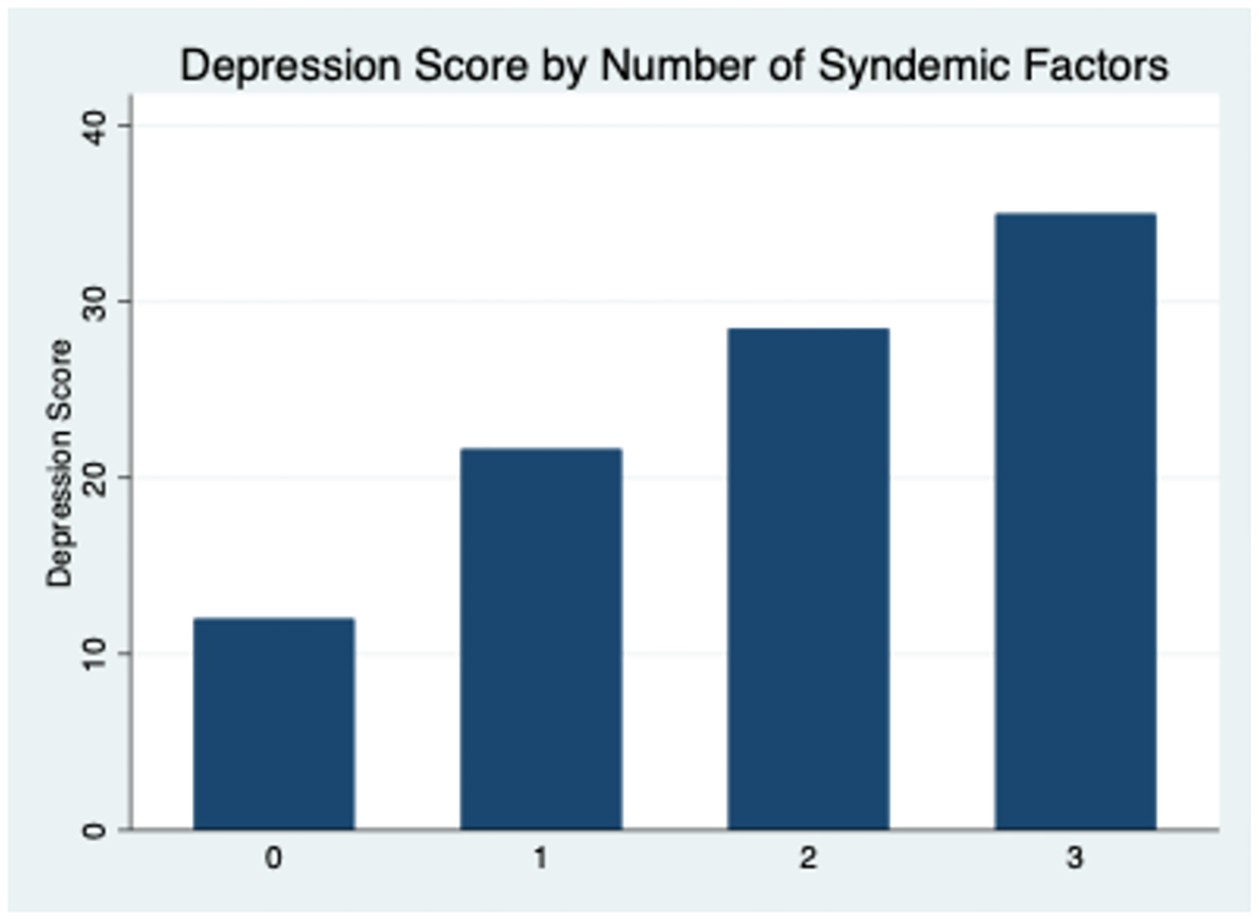
Continuous CESD scores by number of syndemic factors (0–3), among marginally housed and homeless transitional age youth living in San Francisco, CA (N = 100).

The median syndemic score was 2 (IQR = 1, 2), 18% had no syndemic factors, 22% had one, 38% had two and 21% had three (Table 1). Three of three possible distinct associations among the syndemic factors were positive and significant, suggesting a high level of clustering among the syndemic factors examined in this analysis (Table 2). The prevalence of being at risk of clinical depression by the number of syndemic factors was: 27% for zero syndemic factors, 68% for one syndemic factor, 83% for two syndemic factors and 100% for those experiencing three syndemic factors (Table 3). This is also demonstrated in Fig 1 which shows that the number of syndemic factors increases linearly with depression scores (Fig 1).

**Table 2:** Pairwise correlation coefficients between syndemic factors among marginally housed and homeless transitional age youth in San Francisco, CA (N = 100).

	Symptoms of PTSD	*P*	Symptoms of moderate or severe anxiety	*P*
Symptoms of PTSD^1^				
Symptoms of moderate or severe anxiety^2^	0.50	<0.001		
Polysubstance use^3^	0.244	0.015	0.21	0.031

Notes:

^1^The PCL-5 was used to measure symptoms of PTSD using a cutoff of 33.

^2^The GAD-7 was used to measure symptoms of moderate or severe anxiety using a cutoff of 10.

^3^Polysubstance use in the past three months was defined as using three or more drugs excluding alcohol.

**Table 3:** The prevalence of those at risk of clinical depression by number of syndemic factors among marginally housed and homeless transitional age youth in San Francisco, CA (N = 100).

	0	1	2	3
	n = 18 (%)	n = 22 (%)	n = 37 (%)	n = 21 (%)
At risk of clinical depression^1^	5 (28)	15 (68)	31 (84)	21 (100)

Notes:

^1^The CESD-20 was used to measure being at risk of clinical depression using a cutoff of 16.

Results from the bivariate Poisson regression models are presented in Table 4. In adjusted Poisson regression models (1–5), those with symptoms of moderate or severe anxiety in the past two weeks were more likely to be at risk of clinical depression compared to those with minimal or mild symptoms of anxiety (adjusted risk ratio [aRR] = 1.62, 95% confidence interval [CI] = 1.23–2.12, *P*<0.001). TAY experiencing symptoms of PTSD in the past month were significantly more likely to be at risk of clinical depression compared to those without symptoms of PTSD (aRR = 3.78, 95% CI = 1.58–9.04, *P* = 0.003). TAY who engaged in polysubstance use, defined as using three or more distinct drugs (excluding alcohol) in the past three months were also more likely to be at risk of clinical depression compared to those who did not engage in polysubstance use (aRR = 1.33, 95% CI = 1.06–1.68), *P* = 0.012) (Table 4).

**Table 4:** Bivariate and multivariable modified poisson regression models examining factors associated with being at risk of clinical depression among marginally housed and homeless transitional age youth in San Francisco, CA (N = 100).

*Variable*	Unadjusted Relative Risk	95% CI	*P*	Adjusted Relative Risk	95% CI	*P*
Age in years	0.97	(0.91–1.04)	0.537			
*Race/ethnicity*						
White	Reference					
Multiracial	1.04	(0.76–1.43)	0.763			
Black	1.01	0.72–1.43)	0.916			
Other/decline to state	0.91	(0.56–1.47)	0.721			
*Gender*						
Male	Reference					
Female	1.10	(0.86–1.41)	0.437			
Other	1.12	(0.70–1.79)	0.625			
*Sexual orientation*						
Heterosexual	Reference					
Gay, lesbian, bisexual or pansexual	1.16	(0.63–1.02)	0.08			
*Housing Status*						
Stably housed	Reference					
Unstably housed^1^	1.05	(0.79–1.39)	0.73			
Homeless	0.81	(0.63–1.05)	0.12			
Living with HIV	1.00	(0.75–1.32)	0.983			
*Mental Health*						
Symptoms of moderate or severe anxiety^2^	1.66	(1.27–2.18)	<0.001	1.62	(1.23–2.12)	<0.001
Symptoms of post-traumatic stress disorder^3^	3.44	(1.59–7.42)	0.002	3.78	(1.58–9.04)	0.003
High exposure to traumatic events prior to age 18^4^	1.27	(0.89–1.81)	0.174			
*Substance Use*						
Polysubstance use past three months^5^	1.31	(1.05–1.62)	0.014	1.33	(1.06–1.68)	0.012
Binge drinking past year^6^	1.15	(0.86–1.53)	0.331			
*Syndemic variables*						
Continuous syndemic score (0–3), per 1-factor increase	1.39	(1.21–1.59)	<0.001	1.40	(1.21–1.62)	<0.001
Syndemic score of 0	Reference					
Syndemic score of 1	2.45	(1.10–5.47)	0.028	2.68	(1.08–6.65)	0.032
Syndemic score of 2	3.01	(1.40–6.46)	0.005	3.24	(1.34–7.83)	0.009
Syndemic score of 3	3.60	(1.70–7.61)	0.001	3.97	(1.65–9.52)	0.002

Notes:

^1^Participants were considered unstably housed if they were living with a relative or in a group home.

^2^The GAD-7 was used to measure symptoms of anxiety.

^3^The PCL-5 was used to measure symptoms of PTSD using a cutoff of 33.

^4^The ACE’s instrument was used to measure high exposure to traumatic events prior 18 using a cutoff of 4.

^5^ Polysubstance use is defined as using three or more drugs (excluding alcohol).

^6^ Binge drinking was defined as ≥5 drinks for males and ≥4 drinks for females in the same day.

The CESD-20 was used to measure being at risk of clinical depression using a cutoff of 16.

95% CI = 95% confidence interval.

Adjusted models (1–5) controlled for: age in years, gender, race/ethnicity and sexual orientation.

Five adjusted models were built to examine each primary effect separately and each aRR represents the total effect of the primary exposure on depression.

### Syndemic factors and depression

There was a significant linear trend in the relationship between the number of syndemic factors ranging from 0–3 and being at risk of clinical depression, such that for every one-unit increase in the number of syndemic factors, the risk of being clinically depressed increased significantly (aRR = 1.40, 95% CI = 1.21–1.62), *P*<0.001). Similarly, the risk of clinical depression increased by the number of syndemic factors reported; compared to TAY with zero syndemic factors, those with one, two or three syndemic factors were significantly more likely to be at risk of clinical depression: (aRR = 2.68, 95% CI = 1.08–6.65, *P* = 0.032), (aRR = 3.24, 95% CI = 1.24–7.83, *P* = 0.003) and (aRR = 3.97, 95% CI = 1.65–9.52, *P* = 0.002), respectively (Table 4).

## Discussion

This study of the prevalence and correlates of being at risk of clinical depression and the role of syndemic factors among marginally housed and homeless TAY in San Francisco, identified three important findings. First, the prevalence of being at risk of clinical depression was distressingly high among TAY in this study. Second, syndemic-affected TAY (those experiencing two or more syndemic factors) were significantly more likely to be at risk of clinical depression compared to those with fewer syndemic factors. Third, there was a high prevalence of the syndemic factors examined including, symptoms of moderate or severe anxiety, symptoms of PTSD and polysubstance use. These findings may be informative for social and behavioral health services targeting marginally housed or homeless TAY.

Approximately three quarters of TAY in our study were at risk of clinical depression, underlining the need for further psychiatric evaluation in this population [32–36]. Depression among TAY has been shown to increase the risk of suicidality, attempted suicide and problematic substance use [37]. The literature among TAY and other populations including young adults has also established connections between anxiety, PTSD, drug use and depression, showing how these factors tend to co-occur [38–42]. Given the high prevalence of being at risk of clinical depression among TAY in this study, behavioral treatments including, cognitive behavioral therapy and interpersonal psychotherapy [34, 43] may help treat those with depression and those at risk of developing depression.

There was a high level of clustering among the syndemic factors measured in our study, including symptoms of moderate or severe anxiety, symptoms of PTSD and polysubstance use, suggesting that these factors tend to co-occur. Syndemic-affected TAY were more likely to be at risk of clinical depression compared to their counterparts, suggesting that the clustering of these psychosocial problems has an additive effect on the risk of depression among TAY in our study. This is shown in our model examining a nominal syndemic score on the risk of clinical depression, where those with two or three syndemic factors were more likely to be at risk of clinical depression compared to those with zero or one, showing that syndemic-affected TAY may experience worse psychological outcomes compared to their counterparts. These findings are supported by prior research which shows that depression is associated with anxiety and PTSD, co-occurring more frequently than depression occurs alone [44]. It is important to note that co-occurring depression, anxiety and PTSD are also associated with reduced recovery and increased chronicity of illness, compared to experiencing one of these conditions alone [45–47].

To address syndemic risk among marginally housed and homeless TAY, structural interventions including, comprehensive medical, social and behavioral health services that have streamlined processes to treat multiple co-occurring conditions may be helpful [9, 48]. This can be achieved by increasing training programs for primary care and mental health clinicians to maximize the potential for treating mental health co-morbidities (e.g., anxiety, depression, PTSD and substance use disorder). In addition, substance use treatment programs for unstably housed youth which incorporate harm reduction principals like “S*eeking Safety*”, a group-based psychotherapy model that simultaneously addresses substance use and PTSD should be considered [49, 50].

### Limitations

Our study has limitations. The baseline assessment did not include diagnostic interviewing for confirmation of depression, anxiety and PTSD. Therefore, data presented on these mental health conditions are based on survey responses and do not represent official diagnoses. Clinical research leveraging diagnostic tools including neuropsychological or cognitive evaluation conducted by psychiatric partitioners is needed to assess the prevalence of diagnosed cases among homeless and marginally housed TAY. The modest sample size, may have increased our chances of committing a type II error (i.e., failing to detect an association that exists). For instance, it is possible that the association between adverse childhood experiences and being at risk of clinical depression would have been detected with a larger sample. Smaller sample sizes can also lead to wider confidence intervals and imprecise point estimates. However, it should be noted that we used modified Poisson regression with robust error variances to estimate more precise point estimates and smaller confidence intervals. We used cross-sectional data which precludes our ability to draw causal inferences or disentangle temporal associations, thus the present study only reports on associations with being at risk of clinical depression. Other factors that impact the external validity of our study include the use of non-probability sampling methods to recruit the study population. Additionally, because this is an observational study which did not involve randomization to an intervention or control condition, we are not able to rule out potential unmeasured confounders or make any causal inferences from our findings. Finally, we relied on self-reported data of certain sensitive behaviors including drug and alcohol use which may be subject to social desirability bias.

## Conclusions

Despite these limitations, this study provides important insights into the prevalence and correlates of being at risk of clinical depression and the role of syndemic factors among marginally housed and homeless TAY in San Francisco. Findings from this study show that the prevalence of being at risk of clinical depression among TAY in this study is high and driven by the syndemic effects of symptoms of anxiety, symptoms of PTSD and polysubstance use. Thus, this study addresses an important gap in research on how syndemic experiences fuel inequalities in psychological outcomes among marginally housed and homeless TAY in San Francisco, CA.

## References

[1] Morton MH, Dworsky A, Matjasko JL, Curry SR, Schlueter D, Chávez R, et al. Prevalence and Correlates of Youth Homelessness in the United States. J Adolesc Heal Off Publ Soc Adolesc Med 2018;62:14–21. doi: 10.1016/j.jadohealth.2017.10.006 29153445 PMC5826721

[2] Perlman S, Willard J, Herbers JE, Cutuli J j., Eyrich Garg KM. Youth Homelessness: Prevalence and Mental Health Correlates. J Soc Social Work Res 2014;5:361–77. doi: 10.1086/677757 26638508

[3] Edidin JP, Ganim Z, Hunter SJ, Karnik NS. The Mental and Physical Health of Homeless Youth: A Literature Review. Child Psychiatry Hum Dev 2012;43:354–75. doi: 10.1007/s10578-011-0270-1 22120422

[4] Merscham C, Van Leeuwen JM, McGuire M. Mental health and substance abuse indicators among homeless youth in Denver, Colorado. Child Welfare 2009;88:93–110. 19777794

[5] Cochran BN, Stewart AJ, Ginzler JA, Cauce AM. Challenges faced by homeless sexual minorities: Comparison of gay, lesbian, bisexual, and transgender homeless adolescents with their heterosexual counterparts. Am J Public Health 2002;92:773–7. doi: 10.2105/ajph.92.5.773 11988446 PMC1447160

[6] Kamieniecki GW. Prevalence of Psychological Distress and Psychiatric Disorders Among Homeless Youth in Australia: A Comparative Review. Aust New Zeal J Psychiatry 2001;35:352–8. doi: 10.1046/j.1440-1614.2001.00910.x 11437809

[7] Slesnick N, Prestopnik J. Dual and Multiple Diagnosis Among Substance Using Runaway Youth. Am J Drug Alcohol Abuse 2005;31:179–201. doi: 10.1081/ada-200047916 15768577 PMC2430748

[8] Busen NH, Engebretson JC. Facilitating risk reduction among homeless and street-involved youth. J Am Acad Nurse Pract 2008;20:567–75. doi: 10.1111/j.1745-7599.2008.00358.x 19128341

[9] Dawson-Rose C, Shehadeh D, Hao J, Barnard J, Khoddam-Khorasani L, Leonard A, et al. Trauma, substance use, and mental health symptoms in transitional age youth experiencing homelessness. Public Health Nurs 2020;37:363–70. doi: 10.1111/phn.12727 32202664

[10] Salomonsen-Sautel S, Van Leeuwen JM, Gilroy C, Boyle S, Malberg D, Hopfer C. Correlates of Substance Use Among Homeless Youths in Eight Cities. Am J Addict 2008;17:224–34. doi: 10.1080/10550490802019964 18464000

[11] Auerswald CL, Lin JS, Parriott A. Six-year mortality in a street-recruited cohort of homeless youth in San Francisco, California. PeerJ 2016;4:e1909. doi: 10.7717/peerj.1909 27114873 PMC4841235

[12] Whitbeck LB, Simons RL. Life on the Streets: The Victimization of Runaway and Homeless Adolescents. Youth Soc 1990;22:108–25. doi: 10.1177/0044118X90022001007

[13] Stewart AJ, Steiman M, Cauce AM, Cochran BN, Whitbeck LB, Hoyt DR. Victimization and Posttraumatic Stress Disorder Among Homeless Adolescents. J Am Acad Child Adolesc Psychiatry 2004;43:325–31. doi: 10.1097/00004583-200403000-00015 15076266

[14] Reck J. Homeless Gay and Transgender Youth of Color in San Francisco: “No One Likes Street Kids”—Even in the Castro. J LGBT Youth 2009;6:223–42. doi: 10.1080/19361650903013519

[15] Gangamma R, Slesnick N, Toviessi P, Serovich J. Comparison of HIV Risks among Gay, Lesbian, Bisexual and Heterosexual Homeless Youth. J Youth Adolesc 2008;37:456–64. doi: 10.1007/s10964-007-9171-9 18607514 PMC2443720

[16] Singer M, Clair S. Syndemics and public health: reconceptualizing disease in bio-social context. Med Anthropol Q 2003;17:423–41. doi: 10.1525/maq.2003.17.4.423 14716917

[17] Singer M, Bulled N, Ostrach B, Mendenhall E. Syndemics and the biosocial conception of health. Lancet 2017;389:941–50. doi: 10.1016/S0140-6736(17)30003-X 28271845

[18] Singer M. A dose of drugs, a touch of violence, a case of AIDS: Conceptualizing the SAVA Syndemic. Free Inq Creat Sociol 1996;24:99–110.

[19] Singer M, Bulled N, Ostrach B. Whither syndemics?: Trends in syndemics research, a review 2015–2019. Glob Public Health 2020;15:943–55. doi: 10.1080/17441692.2020.1724317 32037962

[20] Street L. Larkin Street n.d.

[21] Center for Epidemiological Studies Depression (CESD) n.d. http://www.apa.org/pi/about/publications/caregivers/practice-settings/assessment/tools/depression-scale.aspx (accessed July 21, 2018).

[22] Weathers FW, Litz BT, Herman DS, Huska JA, Keane TM. The PTSD Checklist (PCL): Reliability, validity, and diagnostic utility. Annu. Conv. Int. Soc. Trauma. Stress Stud. San Antonio, TX, vol. 462, San Antonio, TX; 1993.

[23] Mossman SA, Luft MJ, Schroeder HK, Varney ST, Fleck DE, Barzman DH, et al. The generalized anxiety disorder 7-item (GAD-7) scale in adolescents with generalized anxiety disorder: Signal detection and validation. Ann Clin Psychiatry Off J Am Acad Clin Psychiatr 2017;29:227. 29069107 PMC5765270

[24] Felitti VJ, Anda RF, Nordenberg D, Williamson DF, Spitz AM, Edwards V, et al. Relationship of childhood abuse and household dysfunction to many of the leading causes of death in adults. The Adverse Childhood Experiences (ACE) Study. Am J Prev Med 1998;14:245–58. doi: 10.1016/s0749-3797(98)00017-8 9635069

[25] Boullier M, Blair M. Adverse childhood experiences. Paediatr Child Health (Oxford) 2018;28:132–7.

[26] Group WHOAW. The alcohol, smoking and substance involvement screening test (ASSIST): development, reliability and feasibility. Addiction 2002;97:1183–94. doi: 10.1046/j.1360-0443.2002.00185.x 12199834

[27] Stall R, Mills TC, Williamson J, Hart T, Greenwood G, Paul J, et al. Association of co-occurring psychosocial health problems and increased vulnerability to HIV/AIDS among urban men who have sex with men. Am J Public Health 2003;93:939–42. doi: 10.2105/ajph.93.6.939 12773359 PMC1447874

[28] Jain JP, Strathdee SA, Patterson TL, Semple SJ, Harvey-Vera A, Magis-Rodríguez C, et al. Perceived barriers to pre-exposure prophylaxis use and the role of syndemic factors among female sex workers in the Mexico-United States border region: a latent class analysis. AIDS Care 2019:1–10. doi: 10.1080/09540121.2019.1626338 31163975 PMC6891112

[29] Sullivan KA, Messer LC, Quinlivan EB. Substance abuse, violence, and HIV/AIDS (SAVA) syndemic effects on viral suppression among HIV positive women of color. AIDS Patient Care STDS 2015;29:S42–8. doi: 10.1089/apc.2014.0278 25397666 PMC4283071

[30] Zou G. A modified poisson regression approach to prospective studies with binary data. Am J Epidemiol 2004;159:702–6. doi: 10.1093/aje/kwh090 15033648

[31] Westreich D, Greenland S. The table 2 fallacy: presenting and interpreting confounder and modifier coefficients. Am J Epidemiol 2013;177:292–8. doi: 10.1093/aje/kws412 23371353 PMC3626058

[32] Gattis MN, Larson A. Perceived racial, sexual identity, and homeless status-related discrimination among Black adolescents and young adults experiencing homelessness: Relations with depressive symptoms and suicidality. Am J Orthopsychiatry 2016;86:79. doi: 10.1037/ort0000096 26460699

[33] Medalia A, Saperstein AM, Huang Y, Lee S, Ronan EJ. Cognitive skills training for homeless transition-age youth: feasibility and pilot efficacy of a community based randomized controlled trial. J Nerv Ment Dis 2017;205:859. doi: 10.1097/NMD.0000000000000741 28937497 PMC5679070

[34] Winiarski DA, Rufa AK, Bounds DT, Glover AC, Hill KA, Karnik NS. Assessing and treating complex mental health needs among homeless youth in a shelter-based clinic. BMC Health Serv Res 2020;20:1–10. 10.1186/s12913-020-4953-9 PMC7014693 32046711

[35] Chan V, Moore J, Derenne J, Fuchs DC. Transitional age youth and college mental health. Child Adolesc Psychiatr Clin 2019;28:363–75. doi: 10.1016/j.chc.2019.02.008 31076114

[36] Bender K, Ferguson K, Thompson S, Langenderfer L. Mental health correlates of victimization classes among homeless youth. Child Abuse Negl 2014;38:1628–35. doi: 10.1016/j.chiabu.2014.03.001 24725619

[37] Hatchel T, Ingram KM, Mintz S, Hartley C, Valido A, Espelage DL, et al. Predictors of suicidal ideation and attempts among LGBTQ adolescents: The roles of help-seeking beliefs, peer victimization, depressive symptoms, and drug use. J Child Fam Stud 2019;28:2443–55. 10.1007/s10826-019-01339-2 PMC11452144 39372436

[38] Georgiades K, Boylan K, Duncan L, Wang L, Colman I, Rhodes AE, et al. Prevalence and correlates of youth suicidal ideation and attempts: evidence from the 2014 Ontario Child Health Study. Can J Psychiatry 2019;64:265–74. doi: 10.1177/0706743719830031 30978144 PMC6463356

[39] Shin SH, McDonald SE, Conley D. Patterns of adverse childhood experiences and substance use among young adults: A latent class analysis. Addict Behav 2018;78:187–92. doi: 10.1016/j.addbeh.2017.11.020 29179155 PMC5783745

[40] Anda RF, Whitfield CL, Felitti VJ, Chapman D, Edwards VJ, Dube SR, et al. Adverse childhood experiences, alcoholic parents, and later risk of alcoholism and depression. Psychiatr Serv 2002;53:1001–9. doi: 10.1176/appi.ps.53.8.1001 12161676

[41] Bienvenu OJ, Colantuoni E, Mendez-Tellez PA, Shanholtz C, Dennison-Himmelfarb CR, Pronovost PJ, et al. Co-occurrence of and remission from general anxiety, depression, and posttraumatic stress disorder symptoms after acute lung injury: a 2-year longitudinal study. Crit Care Med 2015;43:642. doi: 10.1097/CCM.0000000000000752 25513784 PMC4336582

[42] Vorspan F, Mehtelli W, Dupuy G, Bloch V, Lépine J-P. Anxiety and substance use disorders: co-occurrence and clinical issues. Curr Psychiatry Rep 2015;17:4. doi: 10.1007/s11920-014-0544-y 25617040

[43] Bender K, Ferguson K, Thompson S, Komlo C, Pollio D. Factors associated with trauma and posttraumatic stress disorder among homeless youth in three U.S. cities: The importance of transience. J Trauma Stress 2010;23:161–8. doi: 10.1002/jts.20501 20146399

[44] American Psychiatric Association., American Psychiatric Association. DSM-5 Task Force. Diagnostic and statistical manual of mental disorders: DSM-5. American Psychiatric Association; 2013.

[45] Fava M, Rush AJ, Alpert JE, Balasubramani GK, Wisniewski SR, Carmin CN, et al. Difference in treatment outcome in outpatients with anxious versus nonanxious depression: a STAR* D report. Am J Psychiatry 2008;165:342–51. doi: 10.1176/appi.ajp.2007.06111868 18172020

[46] Rush AJ, Warden D, Wisniewski SR, Fava M, Trivedi MH, Gaynes BN, et al. STAR* D. CNS Drugs 2009;23:627–47. doi: 10.2165/00023210-200923080-00001 19594193

[47] Rush AJ, Trivedi MH, Wisniewski SR, Nierenberg AA, Stewart JW, Warden D, et al. Acute and longer-term outcomes in depressed outpatients requiring one or several treatment steps: a STAR* D report. Am J Psychiatry 2006;163:1905–17. doi: 10.1176/ajp.2006.163.11.1905 17074942

[48] Sterling S, Weisner C, Hinman A, Parthasarathy S. Access to treatment for adolescents with substance use and co-occurring disorders: challenges and opportunities. J Am Acad Child Adolesc Psychiatry 2010;49:637–46. doi: 10.1016/j.jaac.2010.03.019 20610133 PMC3045032

[49] Jenkins EK, Slemon A, Haines-Saah RJ. Developing harm reduction in the context of youth substance use: insights from a multi-site qualitative analysis of young people’s harm minimization strategies. Harm Reduct J 2017;14:1–11. doi: 10.1186/s12954-016-0127-9 28760146 PMC5537985

[50] Najavits L. Seeking safety: A treatment manual for PTSD and substance abuse. Guilford Publications; 2002. doi: 10.1016/s0740-5472(02)00219-2

